# Near-Infrared Optical Modulation for Ultrashort Pulse Generation Employing Indium Monosulfide (InS) Two-Dimensional Semiconductor Nanocrystals

**DOI:** 10.3390/nano9060865

**Published:** 2019-06-07

**Authors:** Tao Wang, Jin Wang, Jian Wu, Pengfei Ma, Rongtao Su, Yanxing Ma, Pu Zhou

**Affiliations:** College of Advanced Interdisciplinary Studies, National University of Defense Technology, Changsha 410073, China; wangtaobit@163.com (T.W.); wangjin18203@163.com (J.W.); shandapengfei@126.com (P.M.); surongtao@126.com (R.S.); xm_wisdom@163.com (Y.M.)

**Keywords:** indium monosulfide, saturable absorber, mode-locked fiber laser, pulse laser

## Abstract

In recent years, metal chalcogenide nanomaterials have received much attention in the field of ultrafast lasers due to their unique band-gap characteristic and excellent optical properties. In this work, two-dimensional (2D) indium monosulfide (InS) nanosheets were synthesized through a modified liquid-phase exfoliation method. In addition, a film-type InS-polyvinyl alcohol (PVA) saturable absorber (SA) was prepared as an optical modulator to generate ultrashort pulses. The nonlinear properties of the InS-PVA SA were systematically investigated. The modulation depth and saturation intensity of the InS-SA were 5.7% and 6.79 MW/cm^2^, respectively. By employing this InS-PVA SA, a stable, passively mode-locked Yb-doped fiber laser was demonstrated. At the fundamental frequency, the laser operated at 1.02 MHz, with a pulse width of 486.7 ps, and the maximum output power was 1.91 mW. By adjusting the polarization states in the cavity, harmonic mode-locked phenomena were also observed. To our knowledge, this is the first time an ultrashort pulse output based on InS has been achieved. The experimental findings indicate that InS is a viable candidate in the field of ultrafast lasers due to its excellent saturable absorption characteristics, which thereby promotes the ultrafast optical applications of InX (X = S, Se, and Te) and expands the category of new SAs.

## 1. Introduction

Pulse fiber lasers have widespread and important applications in medicine, remote sensing, telecommunication, and material processing, etc. [1,2,3]. In recent years, passively mode-locked fiber lasers based on novel two-dimensional (2D) materials as saturable absorbers (SAs) have attracted much attention. As is widely known, extensive investigations on graphene [4,5,6,7,8,9,10,11] have greatly promoted the exploration of new 2D materials. Starting from the enthusiastic study of graphene, various kinds of materials, such as carbon nanotubes (CNT) [12,13,14,15], transition metal dichalcogenides (TMDs) [16,17,18,19,20,21], topological insulators (TIs) [22,23,24,25,26], and black phosphorus (BP) [27,28,29,30,31], have been employed as SAs and have successfully demonstrated pulse laser operation from visible to mid-infrared optical regions. Recently, III–VI group compounds (MX; M = Ga, In; X = Te, Se, S) have also gained widespread attention and have been applied in the areas of optoelectronic devices, nonlinear optics, ultrafast laser, and terahertz generation due to their large nonlinear effect, high damage threshold, dramatic photo-response and suitable band-gap [32,33,34,35,36,37,38,39,40,41,42,43,44,45]. For example, in 2018, Xu et al. presented a mode-locked laser in the 1 μm region based on InSe as a SA [36]. Other compounds, such as GaS and GaSe, were also shown to be suitable for photo-electric devices [37,38,39,40]. It is obvious that exploration into the practical applications of III–VI group compounds exhibits significance, but is still in its infancy. Thus, it is of necessity to study such compounds for their optical performance in ultrafast laser generation.

Indium monosulfide (InS) is another promising negative (n)-type semiconductor, which belongs to the III–VI group compounds family [41]. InS has been reported to exist in two different crystalline phases—layered and network structural forms—which are connected by fundamental S–In–In–S units [45]. There have also been several studies concerning the optical and electrical properties of InS [41,42,43,44]. The direct and indirect band-gaps of InS are 2.4 eV and 1.9 eV at room temperature, respectively [41,42], which render the InS crystal more sensitive to near-infrared wavelengths than visible wavelengths. InS also has great potential for next-generation flexible and planar devices. In 2016, Ho et al. experimentally generated single crystals of InS micro stripe, which were grown through a physical vapor transport method. Based on these stripe-like InS crystals, a prototype photo-metal-semiconductor field effect transistor (FET) was successfully constructed [44]. However, the nonlinear saturable absorption properties of InS have rarely been explored and are absent from the literature at this stage of research, to the best of our knowledge. It is expected that InS will have comparable optical performance to other reported SAs (such as BP, TIs, TMDs, etc.) due to its suitable band-gap value and typical layered structure.

In this paper, a passively mode-locked Yb-doped fiber laser based on InS-polyvinyl alcohol (PVA) SA was, to our knowledge, demonstrated for the first time. The InS nanosheets were prepared via a liquid-phase exfoliation (LPE) method. The saturation intensity of the InS-SA thin film was measured to be 6.79 MW/cm^2^, while the modulation depth was 5.7%. With the help of this high-quality InS-SA, stable, mode-locked pulses could be observed. The output laser had a pulse width of 486.7 ps at the fundamental cavity frequency of 1.02 MHz. Harmonic mode-locked pulses were also realized by adjusting the polarization states in the cavity. Our results indicated that InS, as an effective SA, could be applied in the field of ultrafast pulse lasers.

## 2. Preparation and Characterization of the InS Sample

**Figure 1:**
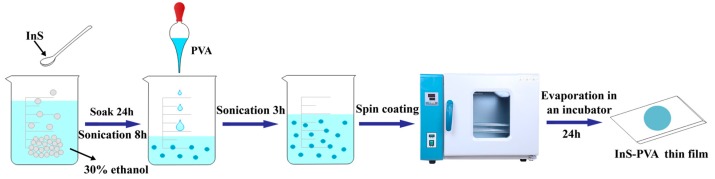
Fabrication process of an indium monosulfide-polyvinyl alcohol (InS-PVA) thin film saturable absorber (SA).

The production process of the InS-PVA SA is shown in Figure 1. In our experiment, the commonly-reported LPE method [36] was employed to prepare the InS solution. This solution was further processed into the film-type InS-PVA SA using the spin-coating method [36]. Firstly, to prepare the InS solution, 50 mg InS powder was added to ethanol solution with a concentration of 30% and soaked for 24 h. Then, the solution was placed in an ultrasonic cleaner for 8 h to carry out liquid-phase exfoliation. Subsequently, the ultrasonic solution was allowed to rest for 1 h and the supernatant was decanted to mix with 4 wt% polyvinyl alcohol (PVA) solution. The mixing ratio was 1:1 by volume. The mixed solution was placed back into the ultrasonic cleaner for 3 h to form a uniform InS-PVA solution. Then, 20 μL of the mixed solution was spin-coated onto the substrate (antistatic glass) and placed in a 20 °C incubator for 24 h. Finally, an InS-PVA thin film was formed, and a 1 × 1 mm^2^ film sample was cut off from the thin film and transferred onto the end face of a fiber ferrule, forming the SA device.

**Figure 2:**
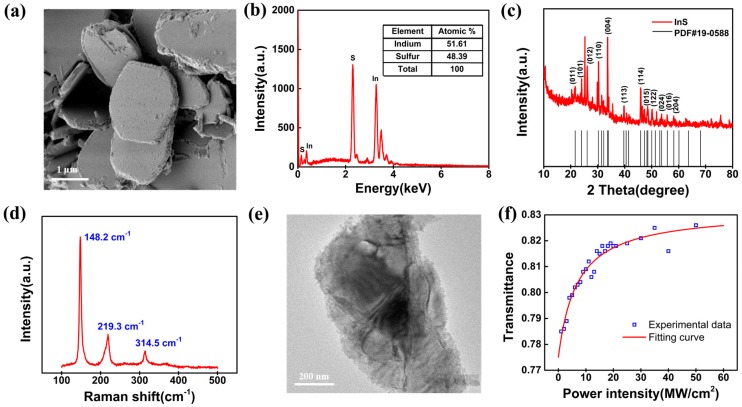
Characteristics of InS crystals: (**a**) A scanning electron microscopy (SEM) image of typical InS flakes; (**b**) energy-dispersive X-ray (EDX) spectroscopy image of the InS; (**c**) X-ray diffraction (XRD) pattern of the InS sample; (**d**) Raman spectroscopy image of the InS; (**e**) a representative transmission electron microscopy (TEM) image of an InS nanosheet after liquid-phase exfoliation; and (**f**) nonlinear transmission of the InS thin film under different incident power intensity.

The characteristics of the prepared InS sample are presented in Figure 2. A scanning electron microscopy (SEM) (Sigma 500, ZEISS, Oberkochen, Germany) image of typical InS flakes is shown in Figure 2a. This measured SEM image shows the typical hexagonal structure of 2D InS flakes, suggesting that the LPE method can be employed to obtain few-layer InS nanosheets. In addition, energy-dispersive X-ray (EDX) spectroscopy was used to characterize the elemental composition of the prepared InS. As shown in Figure 2b, the peaks corresponding to the S and In elements are clear, corresponding with a previous report [42]. In the inset of the figure, the elements In and S are shown to have an atom ratio of 51.61:48.39, which is approximate to 1:1, indicating that the InS flakes had the correct elemental composition and uniform crystal quality. Additionally, X-ray diffraction (XRD) (D8 Advance, Bruker, Billerica, MA, USA) was used to characterize the structure of the prepared InS flakes. As shown in Figure 2c, typical peaks, which match well with standard PDF card #19-0588, were recorded. In particular, the obvious (101) and (004) planes indicated that InS nanosheets with high crystallization quality were prepared in the experiment. As shown in Figure 2d, the InS flakes were also characterized by Raman spectroscopy (LabRAM HR Evolution, Horiba, Kyoto, Japan). Three typical Raman shift peaks located at 148.2 cm^−1^ (In–In stretching mode), 219.3 cm^−1^ (S–In bending mode), and 314.5 cm^−1^ (S–In stretching mode) corresponded with literature data [46,47]. Figure 2e shows a representative transmission electron microscopy (TEM) image (JEM-2100, JEOL, Tokyo, Japan) of the InS solution, which clearly indicates the InS nanosheets had a plicated morphology and an apparent layered crystal structure.

The saturable absorption properties of the InS-PVA thin film were also studied based on the previously reported power-dependent transmission technique [48,49]. The seed laser was a self-made, Yb-doped, mode-locked fiber pulse laser with a central wavelength of 1060.2 nm, repetition rate of 12.36 MHz, and pulse width of 19 ps. The maximum output power was 185 mW after a one-stage amplifier. The nonlinear transmittance of the InS thin film at different incident laser power levels is recorded in Figure 2f. The experimental data were fitted using the following formula [50]:

$$T(I)=1-\frac{\alpha_{s}}{1+I/I_{sat}}-\alpha_{ns}$$

where *T*(*I*) is the optical transmittance, *I* is the incident laser power intensity, *α_s_* is the modulation depth, *I_sat_* is the saturable intensity, and *α_ns_* is the unsaturated loss. The fitted saturation intensity and modulation depth were 6.79 MW/cm^2^ and 5.7%, respectively.

## 3. Experimental Setup and Results

**Figure 3:**
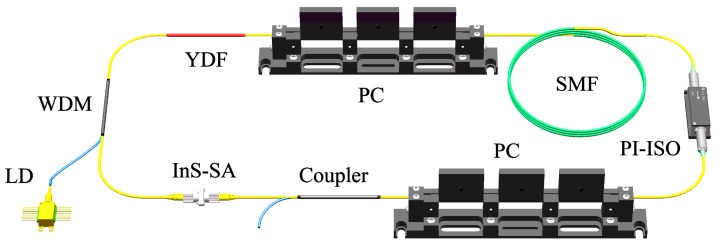
Experimental setup of the mode-locked Yb-doped fiber pulse laser. LD: laser diode; YDF: Yb-doped fiber; SMF: single-mode fiber; WDM: wavelength division multiplexing; PC: polarization controller; PI-ISO: polarization-insensitive isolator; InS-SA: InS saturable absorber.

The experimental setup for the passively mode-locked Yb-doped fiber laser is presented in Figure 3. The gain medium was a 25 cm long Yb-doped fiber (Yb-1200) with a dispersion coefficient of 22 ps^2^/km. The gain fiber was pumped by a single-mode 980 nm laser diode, with a maximum output power of 580 mW, through a broadband wavelength division multiplexing system. In order to ensure unidirectional laser propagation, a polarization-insensitive isolator (PI-ISO) was employed. Two polarization controllers were adopted to optimize the polarization states in the laser cavity. A 10/90 output coupler was adopted to extract the energy from its 10% port. The InS-PVA SA was deposited onto the end face of a fiber ferrule adapter and connected between the coupler and PI-ISO to induce mode-locked operation. The total cavity length was about 206.9 m (0.25 m gain fiber and 206.65 m single-mode fiber with dispersion coefficient of 22 ps^2^/km) and the net dispersion value of this laser cavity was ~4.55 ps^2^. This cavity operated in an all-normal dispersion regime.

**Figure 4:**
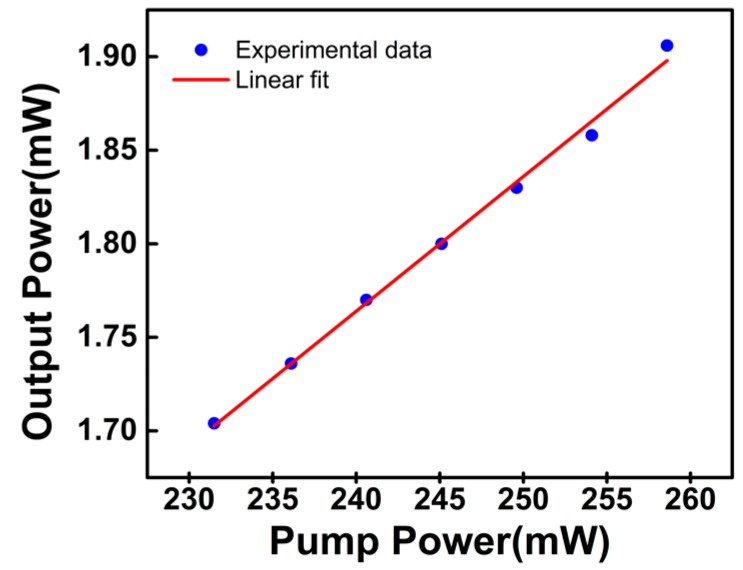
The output power as a function of pump power.

Using the described configuration of the Yb-doped ring fiber laser, which incorporated the InS-based SA, stable mode-locked pulses could be observed when the pump power was increased to 231.5 mW. The threshold power in this work was relatively high compared with previously reported results of other SAs [10,11,20,26]. This high threshold power was probably due to the large cavity loss brought by the devices. The output power increased linearly with increasing pump power, as shown in Figure 4. At the pump power of 258.6 mW, the maximum output power obtained in this work was 1.91 mW. The slope efficiency and optical-to-optical conversion efficiency of this cavity were 7.05% and 0.74%, respectively. Higher conversion efficiency and output power could be expected if an output coupler with a higher output coupling ratio was employed. In future experiments, the cavity parameters should be further optimized to enhance the output performance of the pulse fiber laser.

**Figure 5:**
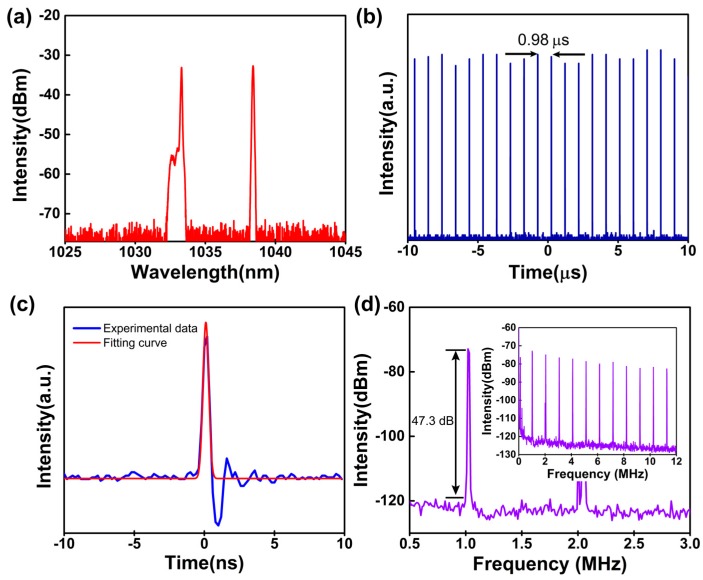
The properties of output mode-locked pulse: (**a**) Spectrum; (**b**) pulse train; (**c**) single pulse profile; and (**d**) radio frequency (RF) spectrum of the mode-locked laser; inset: RF spectrum in a large range of 12 MHz.

Typical pulse properties are shown in Figure 5. Figure 5a depicts the output spectrum of the mode-locked pulse laser. The spectrum has central wavelengths of 1033.3 nm and 1038.4 nm with 3-dB bandwidths of 0.07 nm and 0.10 nm, respectively. The fundamental frequency pulse repeated at the cavity roundtrip time and the repetition rate was 1.02 MHz with a pulse-to-pulse interval of 0.98 μs, as shown in Figure 5b. A magnified single pulse is depicted in Figure 5c. The pulse envelope was further fitted by a Gaussian function and the fitted pulse duration was 486.7 ps. The radio frequency (RF) spectrum of the output pulse was also characterized, as shown in Figure 5d. The signal-to-noise ratio (SNR) was measured to be ~47.3 dB in a small frequency span of 2.5 MHz (0.5–3 MHz) with a resolution of 100 Hz. The RF spectrum in a large range of 12 MHz is presented in the inset of Figure 5d. These results indicated that the pulses obtained in this work had high stability.

**Figure 6:**
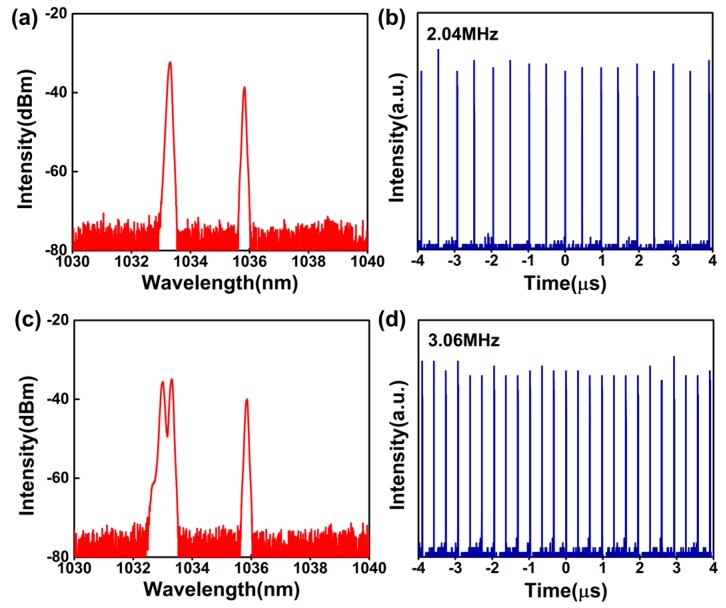
Experimental results for high-order mode-locked pulse: 2nd order (**a**) spectrum and (**b**) pulse train; and 3rd order (**c**) spectrum and (**d**) pulse train.

Besides the aforementioned fundamental frequency mode-locked pulses, harmonic mode-locked pulses could also be observed at a pump power of 236.1 mW. The high-order harmonic mode-locked pulses were achieved by adjusting the polarization controllers (PCs) carefully. Figure 6a,b show the spectrum and pulse train of a 2nd order mode-locked pulse. The spectrum has central wavelengths of 1033.3 nm and 1035.8 nm. The intensity of the 1035.8 nm wavelength was much lower than that of the 1033.3 nm—by about 6.3 dB. Thus, the 2nd harmonic generation actually corresponded to a single emission wavelength. The pulse-to-pulse time was about 0.49 μs, corresponding to the 2nd harmonic frequency. Figure 6c presents the spectrum of a 3rd order mode-locked pulse. The spectrum also has two wavelength components. The top of the 1033 nm wavelength component has a collapse, which is different from the 2nd harmonic operation. In Figure 6d, pulse trains corresponding to the 3rd harmonic pulse are presented. As is known, harmonic pulse generation is the result of a balance between output energy and laser gain. In our experiment, due to the limited pump source and low optical efficiency, no higher harmonic generations were able to be obtained. However, based on a higher pump source and low-loss laser cavity, higher harmonic pulses could be obtained in our future works.

As mentioned, the total laser cavity length was about 206.9 m. Within a long-length laser cavity, self-mode-locked or Q-switched operations are easily observed due to the Kerr effect within the single-mode fiber under high energy [51,52]. In order to verify the effect of the InS-PVA SA on the mode-locked pulse, the sample was removed from the cavity. In this state, as the pump power was increased from 0 to 580 mW, no matter how we adjusted the PCs, no mode-locked pulse was observed, and only continuous wave operation was found. This indicated that the saturable absorption of InS induced the mode-locked pulses. In addition, it was noted that the band-gap of 1.2 eV, corresponding to the mode-locked operation at 1.03 μm in this work, is lower than the band-gap of InS (direct and indirect band-gaps of 2.4 eV and 1.9 eV, respectively), suggesting that sub-band-gap saturable absorption of InS resulted in the observed mode-locked operation. In fact, pulse fiber lasers induced by a sub-band-gap saturable absorption phenomenon have been reported by different groups [35,53,54]. Ideally, perfect crystals have no sub-band-gap absorption, but in a finite crystal structure, sub-band-gap absorption can be observed due to the edge-state [54]. Therefore, the sub-band-gap absorption phenomenon in this work could also be ascribed to edge-state absorption by the InS-PVA SA.

In Table 1, the optical performance parameters of Yb-doped mode-locked lasers based on different SAs are shown. Various mode-locked modulators, including graphene (oxide), CNT, TMD (MoS_2_, WS_2_), TI (Bi_2_Te_3_, Bi_2_Se_3_), BP, InSe and InS, have been employed as SAs successfully in the 1μm region. The pulse laser constructed in this work had similar optical performance to other mode-locked lasers described in the literature. The relatively high pulse threshold of our laser was due to larger intra-cavity loss. Higher output power and a smaller threshold may be expected if the optical devices are further optimized. This work expanded the category of new SAs, indicating that InS is a promising candidate for ultrafast lasers.

## 4. Conclusions

In conclusion, a passively mode-locked Yb-doped fiber pulse laser incorporating an InS-PVA SA was demonstrated experimentally. The nonlinear optical properties of the InS-PVA SA prepared by the LPE method were studied, and the saturation intensity and modulation depth of the InS thin film were 6.79 MW/cm^2^ and 5.7%, respectively. When the pump power was increased to 231.5 mW, stable, mode-locked pulses could be observed, and the maximum output power was recorded as 1.91 mW. The mode-locked laser had a pulse width of 486.7 ps at the fundamental cavity frequency of 1.02 MHz, with a SNR of ~47.3 dB. In addition, harmonic mode-locked pulses were achieved by rotating the PCs in the cavity. These experimental results indicate that InS can be employed as an effective SA for ultrafast laser generation. Considering its many attractive qualities, such as a suitable band-gap and excellent saturable absorption characteristics, it is expected that InS will also find important application in nonlinear optics where an SA material is required.

## Figures and Tables

**Table 1:** Comparison of Yb-doped mode-locked lasers incorporating different SAs.

SA	*α_s_*/*I_sat_*%/MW/cm^2^	Repetition Rate (MHz)	Output Power (mW)	Pulse Width (ps)	Threshold (mW)	SNR (dB)	Reference
Graphene	8/--	0.9	0.37	580	100	>70	10
Graphene oxide	25.31/13.01	14.2	2.1	340	110	65	11
CNT	--	16.37	0.12	4.85	--	60	14
CNT	--	0.177	0.60	1700	--	~50	15
MoS_2_	10.47/0.88 mW	6.74	2.37	656	120	59	20
WS_2_	5.8/	23.26	30	713	550	55	21
Bi_2_Te_3_	1.8/92 W	1.436	0.82	230	200	~77	25
Bi_2_Se_3_	5.2/70 μJ/cm^2^	44.6	33.7	46	153	58	26
BP	8/0.35	13.5	80	--	816	45	31
InSe	4.2/15.6	1.76	16.3	1370	185	45	36
InS	5.7/6.79	1.02	1.91	486.7	231.5	47	Ours

## References

[1] Richardson D., Nilsson J., Clarkson W. (2010). High power fiber lasers: Current status and future perspectives. JOSA B.

[2] Fermann M.E., Hartl I. (2013). Ultrafast fibre lasers. Nat. Photonics.

[3] Nishizawa N. (2014). Ultrashort pulse fiber lasers and their applications. Jpn. J. Appl. Phys..

[4] Bonaccorso F., Sun Z., Hasan T., Ferrari A.C. (2010). Graphene photonics and optoelectronics. Nat. Photonics.

[5] Sun Z., Hasan T., Torrisi F., Popa D., Privitera G., Wang F., Bonaccorso F., Basko D.M., Ferrari A.C. (2010). Graphene Mode-Locked Ultrafast Laser. ACS Nano.

[6] Popa D., Sun Z., Torrisi F., Hasan T., Wang F., Ferrari A.C. (2010). Sub 200 fs pulse generation from a graphene mode-locked fiber laser. Appl. Phys. Lett..

[7] Zhang H., Bao Q., Tang D., Zhao L., Loh K. (2009). Large energy soliton erbium-doped fiber laser with a graphene-polymer composite mode locker. Appl. Phys. Lett..

[8] Wu H., Wu J., Yu Q., Zhang K., Xiao H., Leng J., Xu J., Zhou P. (2017). Over 70 nm broadband-tunable Yb-doped fiber pulse laser based on trilaminar graphene. Laser Phys. Lett..

[9] Wang J., Wang Y., Wang T., Li G., Lou R., Cheng G., Bai J. (2019). Nonlinear Optical Response of Graphene Oxide Langmuir-Blodgett Film as Saturable Absorbers. Nanomaterials.

[10] Zhao L.M., Tang D.Y., Zhang H., Wu X., Bao Q., Loh K.P. (2010). Dissipative soliton operation of an ytterbium-doped fiber laser mode locked with atomic multilayer graphene. Opt. Lett..

[11] Huang S., Wang Y., Yan P., Zhao J., Li H., Lin R. (2014). Tunable and switchable multi-wavelength dissipative soliton generation in a graphene oxide mode-locked Yb-doped fiber laser. Opt. Express.

[12] Hou L., Guo H., Wang Y., Sun J., Lin Q., Bai Y., Bai J. (2018). Sub-200 femtosecond dispersion-managed soliton ytterbium-doped fiber laser based on carbon nanotubes saturable absorber. Opt. Express.

[13] Zhou Y., Lin J., Zhang X., Xu L., Gu C., Sun B., Wang A., Zhan Q. (2016). Self-starting passively mode-locked all fiber laser based on carbon nanotubes with radially polarized emission. Photonics Res..

[14] Hasan T. (2014). Double-Wall Carbon Nanotubes for Wide-Band, Ultrafast Pulse Generation. ACS Nano.

[15] Kelleher E., Travers J., Ippen E., Sun Z., Ferrari A., Popov S., Taylor J. (2009). Generation and direct measurement of giant chirp in a passively mode-locked laser. Opt. Lett..

[16] Niu K., Sun R., Chen Q., Man B., Zhang H. (2018). Passively mode-locked Er-doped fiber laser based on SnS_2_ nanosheets as a saturable absorber. Photonics Res..

[17] Yang Y., Yang S., Li C., Lin X. (2019). Passively Q-switched and mode-locked Tm-Ho co-doped fiber laser using a WS_2_ saturable absorber fabricated by chemical vapor deposition. Opt. Laser Technol..

[18] Liu W., Liu M., OuYang Y., Hou H., Lei M., Wei Z. (2018). CVD-grown MoSe_2_ with high modulation depth for ultrafast mode-locked erbium-doped fiber laser. Nanotechnology.

[19] Luo A., Liu M., Wang X., Ning Q., Xu W., Luo Z. (2015). Few-layer MoS_2_-deposited microfiber as highly nonlinear photonic device for pulse shaping in a fiber laser. Photonics Res..

[20] Du J., Wang Q., Jiang G., Xu C., Zhao C., Xiang Y., Chen Y., Wen S., Zhang H. (2014). Ytterbium-doped fiber laser passively mode locked by few-layer Molybdenum Disulfide (MoS_2_) saturable absorber functioned with evanescent field interaction. Sci. Rep..

[21] Li L., Jiang S., Wang Y., Wang X., Duan L., Mao D., Li Z., Man B., Si J. (2015). WS_2_/fluorine mica (FM) saturable absorbers for all-normal-dispersion mode-locked fiber laser. Opt. Express.

[22] Guo B., Yao Y., Xiao J., Wang R., Zhang J. (2016). Topological Insulator-Assisted Dual-Wavelength Fiber Laser Delivering Versatile Pulse Patterns. IEEE J. Sel. Top. Quantum Electron..

[23] Liu W., Pang L., Han H., Tian W., Chen H., Lei M., Yan P., Wei Z. (2016). 70-fs mode-locked erbium-doped fiber laser with topological insulator. Sci. Rep..

[24] Xu N., Ming N., Han X., Man B., Zhang H. (2019). Large-energy passively Q-switched Er-doped fiber laser based on CVD-Bi_2_Se_3_ as saturable absorber. Opt. Mater. Express.

[25] Chi C., Lee J., Koo J., Lee J.H. (2014). All-normal-dispersion dissipative-soliton fiber laser at 1.06 µm using a bulk-structured Bi_2_Te_3_ topological insulator-deposited side-polished fiber. Laser Phys..

[26] Dou Z., Song Y., Tian J., Liu J., Yu Z., Fang X. (2014). Mode-locked ytterbium-doped fiber laser based on topological insulator: Bi_2_Se_3_. Opt. Express.

[27] Mao D., Li M., Cui X., Zhang W., Lu H., Song K., Zhao J. (2018). Stable high-power saturable absorber based on polymer-black-phosphorus films. Opt. Commun..

[28] Luo Z., Liu M., Guo Z., Jiang X., Luo A., Zhao C., Yu X., Xu W., Zhang H. (2015). Microfiber-based few-layer black phosphorus saturable absorber for ultra-fast fiber laser. Opt. Express.

[29] Xu Y., Jiang X., Ge Y., Guo Z., Zeng Z., Xu Q., Zhang H., Yu X., Fan D. (2017). Size-dependent nonlinear optical properties of black phosphorus nanosheets and their applications in ultrafast photonics. J. Mater. Chem. C.

[30] Wang T., Jin X., Yang J., Wu J., Yu Q., Pan Z., Wu H., Li J., Su R., Xu J. (2019). Ultra-stable pulse generation in ytterbium-doped fiber laser based on black phosphorus. Nanoscale Adv..

[31] Hisyam M.B., Rusdi M.F.M., Latiff A.A., Harun S.W. (2017). Generation of Mode-locked Ytterbium doped fiber ring laser using few-layer black phosphorus as a saturable absorber. IEEE J. Sel. Top. Quantum Electron..

[32] Jana M.K., Pal K., Waghmare U.V., Biswas K. (2016). The Origin of Ultralow Thermal Conductivity in InTe: Lone-Pair-Induced Anharmonic Rattling. Angew. Chem..

[33] Ho C.H., Chu Y.J. (2015). Bending Photoluminescence and Surface Photovoltaic Effect on Multilayer InSe 2D Microplate Crystals. Adv. Opt. Mater..

[34] Sucharitakul S., Goble N.J., Kumar U.R., Sankar R., Bogorad Z.A., Chou F.C., Chen Y.T., Gao X.P.A. (2015). Intrinsic Electron Mobility Exceeding 10^3^ cm^2^/(V s) in Multilayer InSe FETs. Nano Lett..

[35] Yang W., Xu N., Zhang H. (2018). Nonlinear absorption properties of indium selenide and its application for demonstrating pulsed Er-doped fiber laser. Laser Phys. Lett..

[36] Xu N., Yang W., Zhang H. (2018). Nonlinear saturable absorption properties of indium selenide and its application for demonstrating a Yb-doped mode-locked fiber laser. Opt. Mater. Express.

[37] Liu K., Xu J., Zhang X. (2004). GaSe crystals for broadband terahertz wave detection. Appl. Phys. Lett..

[38] Ho C.H., Hsieh M.H., Wu C.C. (2006). Photoconductance and photoresponse of layer compound photodetectors in the UV-visible region. Rev. Sci. Instrum..

[39] Hu P., Wen Z., Wang L., Tan P., Xiao K. (2012). Synthesis of Few-Layer GaSe Nanosheets for High Performance Photodetectors. ACS Nano.

[40] Hu P., Wang L., Yoon M., Zhang J., Feng W., Wang X., Wen Z., Idrobo J.C., Miyamoto Y., Geohegan D.B. (2013). Highly Responsive Ultrathin GaS Nanosheet Photodetectors on Rigid and Flexible Substrates. Nano Lett..

[41] Nishino T., Hamakawa Y. (1977). Preparation and Properties of InS Single Crystals. Jpn. J. Appl. Phys..

[42] Seyam M.A.M. (2001). Optical and electrical properties of indium monosulfide (InS) thin films. Vacuum.

[43] Kushwaha P., Patra A., Anjali E., Surdi H., Singh A., Gurada C., Ramakrishnan S., Prabhu S.S., Gopal A.V., Thamizhavel A. (2014). Physical, optical and nonlinear properties of InS single crystal. Opt. Mater..

[44] Ho C.H., Chen Y.H., Ho J.H. (2016). Optical and photodetector properties of stripe-like InS crystal. RSC Adv..

[45] Hollingsworth J.A., Poojary D.M., Clearfield A., Buhro W.E. (2000). Catalyzed Growth of a Metastable InS Crystal Structure as Colloidal Crystals. J. Am. Chem. Soc..

[46] Gasanly N.M., Özkan H., Aydinli A., Yilmaz İ. (1999). Temperature dependence of the Raman-active phonon frequencies in indium sulfide. Solid State Commun..

[47] Kumaresan R., Ichimura M., Sato N., Ramasamy P. (2002). Application of novel photochemical deposition technique for the deposition of indium sulfide. Mater. Sci. Eng. B.

[48] Guo B., Lyu Q., Yao Y., Wang P. (2016). Direct generation of dip-type sidebands from WS_2_ mode-locked fiber laser. Opt. Mater. Express.

[49] Ming N., Tao S., Yang W., Chen Q., Sun R., Wang C., Wang S., Man B., Zhang H. (2018). Mode-locked Er-doped fiber laser based on PbS/CdS core/shell quantum dots as saturable absorber. Opt. Express.

[50] Garmire E. (2000). Resonant optical nonlinearities in semiconductors. IEEE J. Sel. Top. Quantum Electron..

[51] Nyushkov B.N., Denisov V.I., Kobtsev S.M., Pivtsov V.S., Kolyada N.A., Ivanenko A.V., Turitsyn S.K. (2010). Generation of 1.7-μJ pulses at 1.55 μm by a self-mode-locked all-fiber laser with a kilometers-long linear-ring cavity. Laser Phys. Lett..

[52] Denisov V.I., Nyushkov B.N., Pivtsov V.S. (2010). Self-mode-locked all-fibre erbium laser with a low repetition rate and high pulse energy. Quantum Electron..

[53] Wang S., Yu H., Zhang H., Wang A., Zhao M., Chen Y., Mei L., Wang J. (2014). Broadband Few-Layer MoS_2_ Saturable Absorbers. Adv. Mater..

[54] Woodward R.I., Kelleher E.J.R., Howe R.C.T., Hu G., Torrisi F., Hasan T., Popov S.V., Taylor J.R. (2014). Tunable Q-switched fiber laser based on saturable edge-state absorption in few-layer molybdenum disulfide (MoS_2_). Opt. Express.

